# Carbuncle—Whitlow and Ingrowing of Nails

**Published:** 1877-10-20

**Authors:** O. E. Newton

**Affiliations:** Cincinnati, O.


THE
### SOUTHERN MEDICAL RECORD.

VOL. VII. ATLANTA, GA., OCTOBER 20, 1877. NO. 10.

THOMAS S. POWELL, M.D.,

W. T. GOLDSMITH, M. D.,

R. C. WORD, M. D.,

} EDITORS.

R. C. WORD, Business Manager.

SUBSCRIPTION: TWO DOLLARS PER ANNUM, IN ADVANCE.

☞ All communications, and letters on business connected with THE RECORD for 1877 must be addressed to the Business Manager.

### Original and Selected Articles.

### CARBUNCLE — WHITLOW AND INGROWING OF NAILS.

BY O. E. NEWTON, M.D., CINCINNATI, O.

By the term carbuncle you are to understand a malignant boil, the tendency of which is to become rapidly worse; will be checked only by the proper treatment. Its natural tendency is to grow larger by destruction of adjunct tissue; therefore, the emolient or poulticing treatment indicated in common boils is, when applied, only aggravating to this disease.

The principal distinctive difference between a carbuncle and boil is, when a boil has ripened, generally, one opening is formed, and contains a core which, when sloughed out, or removed, leaves a healthy base, and will soon heal by simple means; while a carbuncle will open with sieve-like openings, instead of one, each forming a distinct sinus, or ulcer, which will eat and destroy, without receiving the proper treatment, which is caustic or the knife.

I treat these cases by destroying and disorganizing, with the caustic of chloride of zinc paste, the diseased tissue, and sloughing it out. Very frequently the whole diseased tissue will come out down to the bone over which the carbuncle has made its appearance. These cases occur most commonly upon the back of the neck. You must not trifle with these cases with *mild, emolient means*, or you may lose your patient. Strike deep and thorough for a total destruction of the whole diseased tissue, and thereby control the disease by the only means, using caustic or the knife.

AN ESPECIAL CASE IN ILLUSTRATION.

Mr. M. T. Ligur, (northern residence No. 142 Clark street, Cincinnati, O.,) a French Southern planter, at Cypremot's Landing, St. Mary's parish, La.

History: Being on his plantation in Louisiana in 1861, was prevented by the war from joining his family, who were on their usual visit North at the outbreak of the war, for four years. During the progress of the war he had been living almost like a hermit, being repeatedly stripped by passing armies, and being often obliged to live upon the coarsest and lowest diet. Upon being notified of the removal of the blockade, he at once started on his way North, going by the way of New Orleans to take boat, where, for the first time, he had an opportunity to satisfy his appetite. He, naturally being a high liver, being a Frenchman, at once began drinking wine, and using the high-seasoned steamboat fare, which he continued to use on his way to Cincinnati. When he reached here he was the subject of a rash, covering his whole body, of a nettle rash character; and eresypelatous inflammation showed itself upon a line of the back of the neck, extending itself over the spinal column. This was the appearance of the external surface when I was called, with a *puffy condition of the skin,* and areolar tissue underneath. The parts at once showed signs of a carbuncle; mortification and sloughing at once followed. The following are the parts that were affected: The back of the head, commencing at a line drawn from ear to ear, and extending downward to the last cervical vertebra, where there seemed to be a line of demarkation between it and the sound flesh, leaving a healthy space of about four inches, from whence the *puffy condition extended to the eighth dorsal vertebra,* where there was another space of about three inches of healthy tissue, from which again the same diseased condition extended to the third lumbar vertebra.

Before any openings had appeared in any of the places, the whole surface of the above described parts showed the probable collection of matter under the skin, there being distinct fluctuation by pressure over those spots. I at once applied a plaster of chloride of zinc paste, covering as much surface as he could stand the destruction of, at one time, over which I applied, every three hours, a poultice of flax seed and pounded charcoal, mixed with brewer's yeast and water. This process of disorganizing by means of the caustic, and favoring suppuration by means of the antiseptic poultices, was continued until the cavities containing the pus were reached.

Having disorganized all the unhealthy tissue and sloughed it out, I dressed it with equal parts of ung. zinc, oxidi. comp., and ung. plumbi comp. The parts excavated to the bone, covering a space of over eighteen inches of surface. So extensive were the parts to be dressed it required from one to one and a half pounds of ointment at each dressing.

I might say, as soon as the caustic was applied, it at once stopped any further extension of the disease. Though the treatment was very radical and painful, it was successful in curing my patient, and gave full satisfaction; and I fully believe, had not this sanguinary caustic treatment been used, my patient would have died.

I was six weeks in attendance, twice per day.

### WHITLOW OR FELON.

This is one of those cases in minor surgery which calls for a correct judgment, and prompt treatment, as it is one of the most painful small difficulties that the human system is called upon to bear. The inflammation is nearly always upon the first phalanx of the finger or thumb. Often when first seen the cellular tissue will be swollen even to severe tightness of the skin, which will be of a bright red color. Finally matter will be formed underneath, and here comes the danger and the indication requiring surgical interference. At the first commencement, it is said that the frequent immersion in scalding alkali, and the part wrapped up in a tight bandage has checked the inflammation. Sometimes the full and frequent painting with Tr. Iodine, the placing it into a lemon cut open, has dispersed the swelling. But it is seldom a physician's attention is called prior to the formation of matter, though not opened, and that is the indication that a cut is to be made from the first joint, down and *through the periosteum to the bone,* extending to the end of the phalanx, which *in every instance* will give prompt relief. The danger growing out of this not being done is: If you wait until the abscess opens of itself the probabilities are that you will have a case of death of the first phalanx, or necrosis. In cutting, be careful to go

*through the periosteum from the point of starting to where you stop.*

After cutting, simply place a pledget of lint or cotton in the opening to keep the edges from uniting—this only to be kept there the first day, over which apply an elm bark poultice. In two days apply the Mayer's Ointment until healed.

I have four cases of necrosis in my surgical cabinet where I performed amputation, and each case could have been prevented if the *early opening had been made.*

### INGROWING OF NAILS.

This is apparently a very trifling case in minor surgery, but you will often be consulted about this kind of trouble, and you will be successful in relieving it by the treatment I give you.

First, soak the toe in hot water, well, to soften the nail as much as possible, then scrape the centre with a piece of glass or a scalpel, and cut off the sharp corners of the ingrowing nail; then anoint cotton in carbolic acid ointment, and crowd it tightly under each corner, with a view of *raising* them out of the *fissure* in the fleshy part around the nail—this cotton to be taken out from time to time and replaced by a new pledget. Allow the nail to grow out at the ends well, to be cut off square—keeping the centre thin, and the edges pressed upwards. If there be, as you will sometime find, a fungus formed on each side of the nail, you will first have to destroy this, with a drop of nitric acid, or a little chloride of zinc paste, before putting the cotton under the nail. This course, sometimes modified according to circumstances, has fully met the demands in these cases.

I have cured such cases as these, who had submitted to the old plan of removing the entire nail the second time, in which cases, the nail came in just where the one had been that was pulled out. *The pulling out of the nail, or cutting off of the corners alone, will not cure these cases.*

